# Identification of Candidate Genes for Seed Glucosinolate Content Using Association Mapping in *Brassica napus* L.

**DOI:** 10.3390/genes6041215

**Published:** 2015-11-18

**Authors:** Cun-Min Qu, Shi-Meng Li, Xiu-Jian Duan, Jin-Hua Fan, Le-Dong Jia, Hui-Yan Zhao, Kun Lu, Jia-Na Li, Xin-Fu Xu, Rui Wang

**Affiliations:** 1 Chongqing Engineering Research Center for Rapeseed, College of Agronomy and Biotechnology, Southwest University, Tiansheng Road 2, Beibei, Chongqing 400716, China; E-Mails: qucunmin@swu.edu.cn (C.-M.Q.); li_shimeng90@163.com (S.-M.L.); duanxiujian9077@163.com (X.-J.D.); lingfeng9511@126.com (J.-H.F.); jiald2014@foxmail.com (L.-D.J.); 13639767407@163.com (H.-Y.Z.); drlukun@swu.edu.cn (K.L.); ljn1950@swu.edu.cn (J.-N.L.); xinfuxu@126.com (X.-F.X.); 2 Engineering Research Center of South Upland Agriculture of Ministry of Education, Southwest University, Beibei, Chongqing 400716, China; 3 Food and Bioproduct Science, University of Saskatchewan, 51 Campus Drive, Saskatoon, SK S7N 5A8, Canada

**Keywords:** *Brassica napus* L., seed glucosinolate (GS) content, association mapping, single nucleotide polymorphism (SNP)

## Abstract

Rapeseed contains glucosinolates, a toxic group of sulfur-containing glucosides, which play critical roles in defense against herbivores and microbes. However, the presence of glucosinolates in rapeseed reduces the value of the meal as feed for livestock. We performed association mapping of seed glucosinolate (GS) content using the 60K *Brassica* Infinium single nucleotide polymorphism (SNP) array in 520 oilseed rape accessions. A total of 11 peak SNPs significantly associated with GS content were detected in growing seasons of 2013 and 2014 and were located on *B. napus* chromosomes A08, A09, C03, and C09, respectively. Two associated regions of GS content covered by these markers were further verified, and three *B. napus* homologous genes involved in the biosynthesis and accumulation of GS were identified. These genes were multigene family members and were distributed on different chromosomes. Moreover, two genes (*BnGRT2* and *BnMYB28*) associated with GS content were validated by the qRT-PCR analysis of their expression profiles. The further identification and functionalization of these genes will provide useful insight into the mechanism underlying GS biosynthesis and allocation in *B. napus*, and the associated SNPs markers could be helpful for molecular maker-assisted breeding for low seed GS in *B. napus*.

## 1. Introduction

Glucosinolates, a group of sulfur-containing glucosides, are secondary metabolites uniquely detected in *Brassicaceae* and provide protection against plant pest and pathogens. However, Glucosinolates also impart a pungent flavor and inhibit thyroid function, resulting in liver and kidney abnormalities if consumed in excess in higher animals [1]. Therefore, it is desirable to reduce the glucosinolate content of seeds, but to maintain a high level of glucosinolates in other tissues to prevent herbivore damage and pathogenic microbes. Rapeseed (*Brassica napus* L., genome AACC, 2n = 38) is an important source of edible oil for humans and protein for live-stock [2]. Therefore, selecting double-low rapeseed, *i.e.*, with seeds lacking erucic acid (another toxic compound produced by rapeseed) and containing only low levels of glucosinolate in seeds, has been an important objective of rapeseed breeding programs globally.

To date, quantitative trait locus (QTL) mapping based on molecular markers is an effective method for identifying candidate alleles involved in a complex trait, by which method numerous markers associated with total GS content have been mapped using different populations of *B. napus* [3,4,5,6,7]. QTLs associated with total GS content were identified on *B. napus* chromosomes N9, N12, and N19 [3,4,5,8,9]. Recently, association mapping, which uses diverse populations to identify associations between allele frequencies and phenotypic variation, has also been used to pinpoint specific genes associated with a trait of interest in plants [10,11,12,13], and has significantly increased the precision of QTL mapping [14]. Using association mapping, 52 simple sequence repeat (SSR) markers were linked to genes involved in the production of phenolic compounds, of these SSRs five were QTL-linked markers in rapeseed [15]. Additionally, 27 SSR markers were found to be associated with variation in seed oil content in both traditional and new-type *B. napus* populations [16]. Although some clusters of single-nucleotide polymorphisms (SNPs) highly associated with the GS content has also been identified in rapeseed in recent years [17,18,19], they need to be verified by the further research.

Recently, the multinational *Brassica* Genome Project [20] has made considerable progress in re-sequencing the *Brassica* genome using next-generation high-throughput DNA sequencing techniques and has developed the *Brassica* 60K SNP BeadChip Array with 52,157 Infinium Type II SNP loci (Isobel Parkin, Agriculture and AgriFood Canada). In this study, we used a panel of 520 rapeseed lines for association mapping of total GS content using the 60k *Brassica* Infinium SNP array. The objective of our study was to identify the consensus association peaked SNP markers, which could be used for molecular marker assisted breeding of low GS content. Meanwhile, we also conducted comparative association mapping with *A. thaliana* to identify candidate genes for GS content in *Brassica* species. This work would lay the foundation for understanding the biosynthesis and accumulation of seed glucosinolate in rapeseed.

## 2. Results

### 2.1. Phenotypic Variations of GS Content

**Figure 1:**
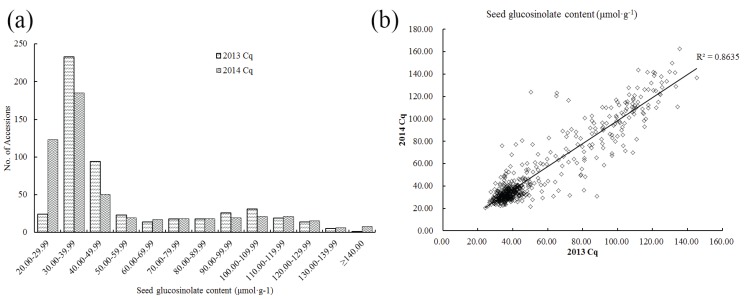
Variations of total seed glucosinolate content of the population (consisting of 520 accessions) in 2013 and 2014. (**a**) Frequency distribution of total seed glucosinolate content; (**b**) Comparison of seed glucosinolate content in 2013 and 2014. Cq refers to the growing region, Chongqing, China.

The total GS content of 520 rapeseed accessions (see Supplementary Material, Table S1 on the journal’s website) were measured with three repeats using near infrared reflectance spectroscopy (NIRS), and the phenotypic variation recorded were observed (Figure 1 and Table 1). In 2013, the GS content ranged from 24.22 to 145.24 μmol·g^−1^ with an average of 54.90 μmol·g^−1^, while in 2014, it ranged from 20.53 to 162.51 μmol·g^−1^ with an average of 52.12 μmol·g^−1^. The high coefficient of variation (Table 1) indicates that there is wide variation in glucosinolate content in this panel of accessions. In addition, approximately 70% of the seeds had low GS content (20.00 to 50.00 μmol·g^−1^, Figure 1a) with a correlation coefficient of 0.8635 in both years (Figure 1b), suggesting that breeding new accessions with a low GS content has long since been a prerequisite for rapeseed cultivation.

**Table 1:** Statistical analysis of seed glucosinolate content in 520 rapeseed accessions.

Year	Range (μmol·g^−1^)	Average (μmol·g^−1^)	Standard Deviation	Coefficient of Variation (%)	Skewness	Kurtosis
2013 Cq (Chongqing)	24.22–145.24	54.90 ± 1.28	29.11	53.02	1.24	0.15
2014 Cq (Chongqing)	20.53–162.51	52.12 ± 1.41	32.24	61.86	1.34	0.56

### 2.2. Population Structure, Relative Kinship and Diversity Panel Analysis

**Figure 2:**
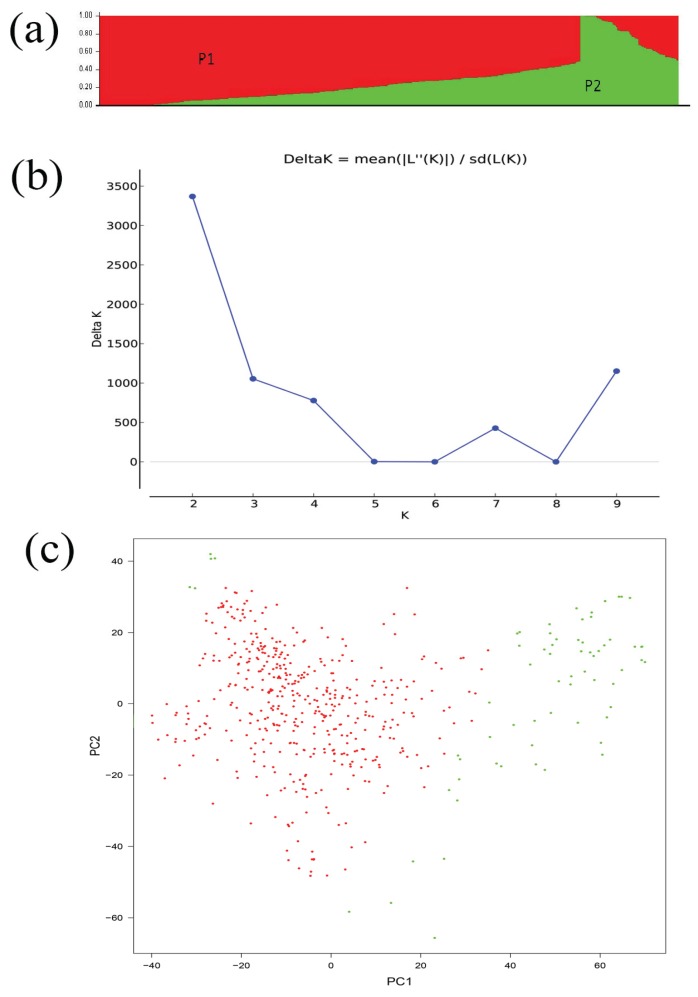
Analysis of the population structure of 520 rapeseed accessions. (**a**), Model-based Bayesian clustering performed by STRUCTURE 2.1 for K = 2 subpopulations (Red indicates subpopulation P1 genotypes and green represent subpopulation P2); (**b**), *ΔK* based on rate of change of *LnP (K)* between successive *K* values; (**c**), The sub-populations in a population of 520 *B. napus* accessions, suggested by Principal Coordinate Analysis (PCA).

A set of SNP markers developed from the *Brassica* 60 K SNP BeadChip Array covering the *B. napus* genome were used to perform association analysis of seed glucosinolate content. Out of a total of 51,264 SNPs to the *B. napus* genome obtained from the blast analysis, 17,131 SNPs were excluded with the call frequency < 90% and an minor allele frequency (MAF) < 0.05, with 34,103 polymorphic SNPs were used for the association analysis. The population structure of 520 rapeseed accessions were classified using by STRUCTURE 2.1 with the optimal value of *K* = 2 (Figure 2a,b) and *Ln P (D)* = −5,240,293.4 (Table 2). However, subpopulation 1, accounting for 89% accession lines, is mainly composed of those from China, whereas subpopulation 2 included the rest of the accessions from a more diverse origin (Figure 2c).

**Table 2:** Average logarithm of the probability of data likelihoods (*LnP*(*D*)), their standard deviations, and *Delta K* for simulations of different *K*-values of the 520 *B. napus* accessions.

K	Mean LnP(K)	Stdev LnP(K)	Delta K
1	−5591550.233	14.608331	—
2	−5240293.4	49.596068	3368.547254
3	−5056103.267	48.066863	1053.798744
4	−4922565.933	57.973902	777.935099
5	−4834128.533	3719.934136	2.630127
6	−4755475.033	8254.854459	0.036322
7	−4677121.367	90.730884	427.251798
8	−4637532.633	2440.407122	0.025228
9	−4597882.333	1000.043131	1151.624163
10	−5709905.867	1989293.383	—

**Figure 3:**
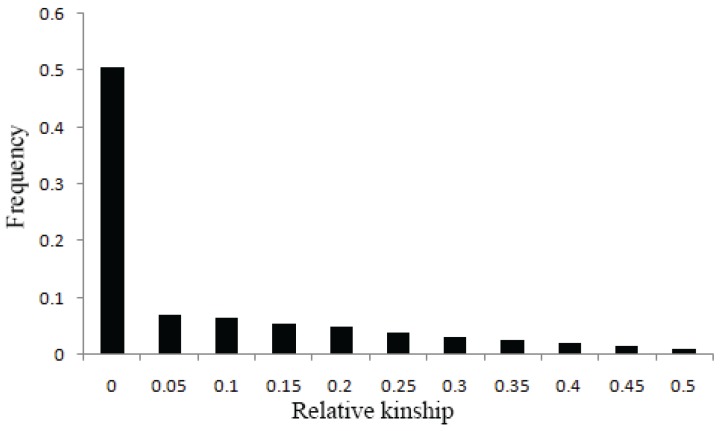
Distribution of relative kinship coefficient across the 520 accessions. Only kinship coefficients of 0 to 0.5 are shown.

Additionally, relative kinships among 520 accessions were estimated using TASSEL 5.2.1 [21], and the results showed that 74.53% of kinship coefficients between lines ranging from 0 to 0.05, and 50.55% were equal to 0 (Figure 3), suggesting that most lines have no kinship or a relatively weak kinship and that spurious associations were controlled.

### 2.3. Association Mapping Analysis

**Figure 4:**
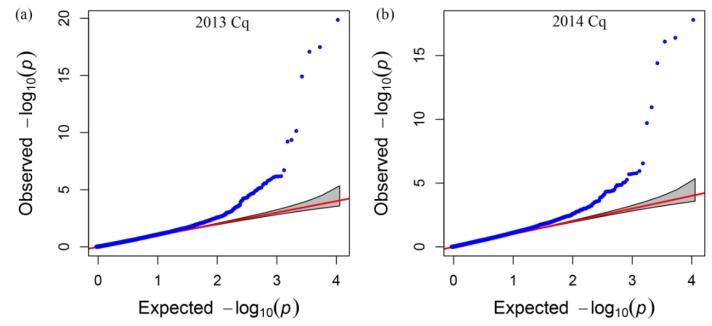
Quantile–quantile plots of evaluated −*log10(P)* from association analysis of seed glucosinolate content using the Q + K model. (**a**) Quantile–quantile plots of evaluated −*log10(P)* from association analysis of seed glucosinolate content in 2013; (**b**) Quantile–quantile plots of evaluated −*log10(P)* from association analysis of seed glucosinolate content in 2014. Cq refers to the growing region, Chongqing, China.

**Figure 5:**
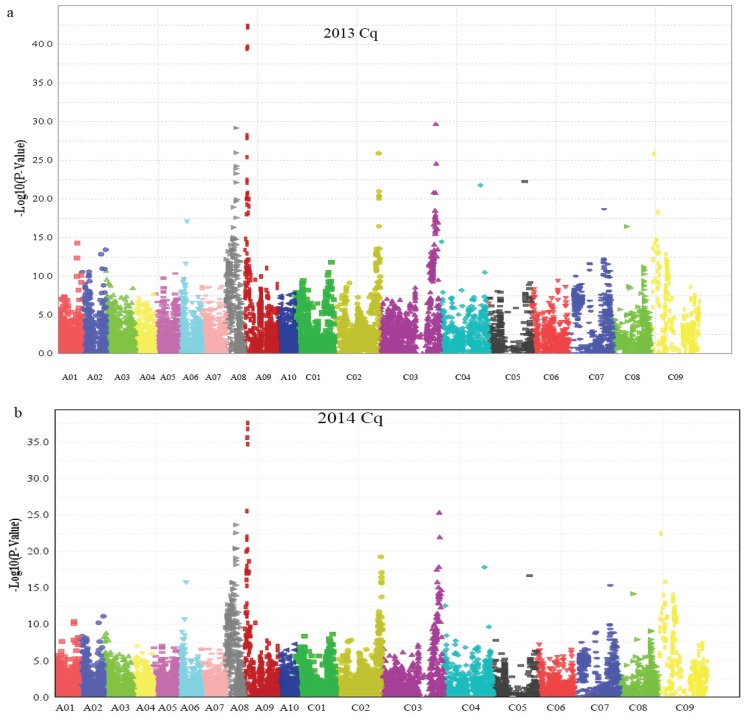
Manhattan plot describing marker-trait associations for the total seed glucosinolate content of the population (consisting of 520 accessions) in 2013 and 2014. (**a**) Manhattan plot describing marker-trait associations for the total seed glucosinolate content in 2013; (**b**) Manhattan plot describing marker-trait associations for the total seed glucosinolate content in 2014. Cq refers to the growing region, Chongqing, China.

After filtering all of the SNP data, we performed genome wide association analysis using the mixed-linear model (MLM) (Q + K) model, and the QQ plot as shown in Figure 4. A total of 15 SNP markers showed significant association with glucosinolate content in the seeds of plants grown in 2013 and 2014 (Table 3), which were significantly associated with the Best Linear Unbiased Prediction (BLUP) value by the PCA model (Figure 5a,b). All markers associated with each trait and the determined phenotypic variation of these markers are listed in Table 3. Interestingly, 11 of the 15 SNP markers were detected in the seeds of plants grown both in 2013 and 2014. Moreover, these 11 SNP markers were located on chromosomes A08, A09, C03, and C09, which had two, six, two, and one SNP marker, respectively. Meanwhile, we detected seven SNP markers with significant associations with glucosinolate content, which were mapped on chromosomes A09 and C09, respectively (Figure 5 and Table 3), indicating that existing homologous loci in this region influenced GS content in *B. napus*. Moreover, the Bn-A09-p3029767 marker, which showed the most significant association with glucosinolate content and which was mapped on chromosome A09, accounted for 33.50% and 31.59% of the phenotypic variance in GS content in samples obtained in 2013 and 2014, respectively (Table 3). Hence, we supposed that the flanking regions of seven peak SNP markers should be associated with candidate genes of GS content in *B. napus* chromosomes A09 and C09, which could be the loci homologous with those of the previous research reports [4].

**Table 3:** Genome-wide significant association signals of seed glucosinolate content.

SNP	*p*-Value		Phenotypic Variation (%)		Chr.	Physical Interval (bp)
	2013	2014	2013	2014		
Bn-A09-p3029767	7.76 × 10^−37^	8.16 × 10^−33^	33.50	31.59	A09	2,949,846–3,135,091
Bn-A09-p3116738	2.61 × 10^−36^	4.05 × 10^−32^	32.94	30.82	A09	
Bn-A09-p3053532	1.79 × 10^−33^	4.94 × 10^−30^	29.96	28.54	A09	
Bn-A09-p3234323	3.15 × 10^−18^		15.19		A09	
Bn-A01-p9125819	2.86 × 10^−32^	9.68 × 10^−30^	28.73	28.23	A09	2,450,781–2,472,858
Bn-A01-p9149601	4.04 × 10^−22^	3.93 × 10^−20^	18.78	18.27	A09	
Bn-A08-p12660208	3.41 × 10^−23^	5.80 × 10^−21^	18.95	18.22	C03	56,050,681–56,466,188
Bn-A08-p12905848	6.81 × 10^−20^	4.05 × 10^−18^	16.71	16.28	C03	
Bn-A09-p1832760	8.94 × 10^−21^	1.89 × 10^−20^	17.52	18.58	A09	2,101,520–2,206,660
Bn-A09-p1727915	5.27 × 10^−20^		16.81		A09	
Bn-scaff_19783_1-p327775	1.91 × 10^−20^	3.39 × 10^−19^	17.22	17.34	C09	2,815,377–2,815,426
Bn-A08-p12913949	1.31 × 10^−20^	1.08 × 10^−18^	16.57	16.01	A08	10,587,677–10,694,560
Bn-A08-p12820786	1.46 × 10^−21^	1.99 × 10^−18^	18.26	16.58	A08	
Bn-scaff_17119_1-p84986	6.12 × 10^−20^		15.96		A08	
Bn-scaff_17177_1-p546184	6.61 × 10^−19^		15.81		C02	44,655,688–44,655,731

### 2.4. Identification and Validation of Candidate Genes

To identify the candidate genes, the *B. napus* genomic sequences between the adjacent peak SNPs were extracted. As a result, the associated regions with glucosinolate content on chromosome A09 and C09 included three *Arabidopsis* homologous genes (*AT5G62680*, *AT5G60890*, and *AT5G61420*) that were involved in the glucosinolate metabolic pathway (Table 4). Homologous genes *AT5G62680* (*GRT2*) and *AT5G60890* (*MYB34*) were approximately 0.2 and 0.1 Mb away from the significant SNP, Bn-A09-p3116738 and Bn-A09-p3029767 on chromosome A09, respectively; and the homologous gene *AT5G61420* (*MYB28*) was 0.3 Mb away from the significant SNPs, Bn-scaff_19783_1-p327775, on chromosome C09 (Table 3). In addition, the association region of GS content was also detected on chromosome A08, maybe in accordance with Xu *et al.* [7]. These results suggested that association analysis is effective for identifying candidate genes by comparing the genomic sequences of the peak SNPs. *B. napus* was derived from interspecific hybridization of *B. rapa* and *B. oleracea* to form an allotetraploid species, and major gene loss is typical after polyploidy formation in eukaryotes [22]. Each of the orthologous blocks corresponding to ancestral blocks could be identified using collinearity between orthologues on the genome of *B. rapa*, *B. oleracea*, *B. napus*, and *A. thaliana* genome. The copies of each gene were identified and distributed in LF, MF1, MF2 and non-genome of *B. rapa*, *B. oleracea* and *B.napus* (Table 4). These results provide a chance to study gene retention in triplicated genomes.

**Figure 6:**
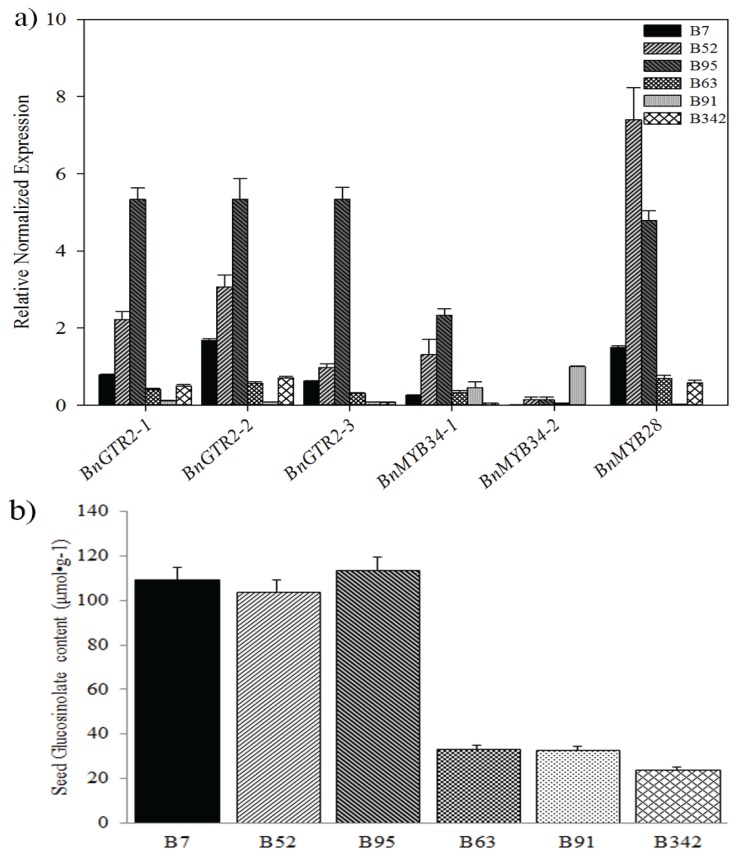
Relative expression of the candidate genes and seed glucosinolate content of *B. napus* accessions. (**a**) Relative expressions of the candidate genes detected by qRT-PCR. The expression levels were normalized with *BnActin7* and *BnUBC21* genes. Error bars indicated the SE for three independent experiments. The primers are listed in Supplementary Material, Table S2 on the journal’s website. (**b**) Means and standard error of seed glucosinolate content of six *B. napus* accessions in 2013 and 2014.

**Table 4:** The different copies of candidate genes in the *B. rapa*, *B. oleracea* and *B. napus* genome.

Species	Lf ^a^	MF1 ^b^	MF2 ^c^	Non-Genome Triplication ^d^	AGI NO.	Description
*Bra*	Bra010111	Bra029248	Bra035886	Bra035885	AT5G62680	GLUCOSINOLATE TRANSPORTER-2
*Bol*	Bol019440	Bol020699	Bol019185			
*BnaA*	BnaA06g22160D	BnaA02g33530D	BnaA09g06190D			
*BnaC*	BnaC03g51560D	BnaC02g42260D	BnaC09g05810D	BnaC02g42280D		
*Bra*	Bra013000	Bra029350	Bra035954		AT5G60890	myb domain protein 34 (MYB34)
*Bol*	Bol017062	Bol007760	Bol036264			
*BnaA*	BnaA03g39790D		BnaA09g05480D	BnaAnng06630D		
*BnaC*	BnaC02g41860D	BnaC09g05060D	BnaCnng21270D			
*Bra*	Bra012961	Bra029311	Bra035929		AT5G61420	myb domain protein 28 (MYB28)
*Bol*	Bol017019	Bol007795	Bol036286			
*BnaA*	BnaA03g40190D					
*BnaC*		BnaC09g05300D	BnaCnng43220D			

^a^ LF indicates the least fractionated blocks; ^b^ MF1 indicates the medium fractionated blocks; ^c^ MF2 indicated the most fractionated blocks; ^d^ Non-genome triplication indicates that these copies are not triplicated genome segments; *Bra*: *B. rapa*; *Bol*: *B. oleracea*; *BnaA*: *B. napus A* genome; *BnaC*: *B. napus C* genome.

The qRT-PCR analysis showed that *BnGTR2* and *BnMYB28* had higher expression levels in relatively high GS content than in relative low GS content lines (Figure 6a,b). This result confirms strong association of *BnGTR2* and *BnMYB28* with GS content in *B. napus*, suggesting that they could play an important role in the glucosinolate biosynthesis in *B. napus*. Therefore, functional characterization of candidate genes will provide more clues so as to illuminate the molecular mechanism of glucosinolate in *B. napus*.

## 3. Discussion

In *B. napus*, selecting double-low seed lines with zero seed erucic acid and low GS content is an important breeding goal. QTL mapping has been used to map genes involved in rapeseed glucosinolate content, and the substantial evidence showed that total GS content is controlled by at least 4 to 5 gene loci in *B. napus* [3,4,5,6,8,9]. Association analysis has become a popular method for QTL mapping, as it offers a high throughput and high resolution means of identifying novel and superior alleles [23], and has been successfully used in *B. napus* in past two years [18,19,24,25,26]. In addition, high density SNPs identified in *B. napus* facilitating linkage disequilibrium (LD) studies can be used to identify rapeseed genes underlying seed glucosinolate content by comparing the genomics datasets of *Brassica* and *Arabidopsis* [17].

In this study, we identified 15 SNP markers with significant associations to GS content using the *Brassica* 60 K SNP BeadChip Array. Of which, 11 showed high LD to this trait in both years which were distributed on chromosomes A08, A09, C03, and C09, respectively (Table 3). Several previous studies detected major QTLs for seed glucosinolate content on chromosomes A09 and C09 [3,4,5,6,7,8,9]. For example, one QTL region for GS was mapped between the markers SNP19550A09 and SNP20943A09 [6], while other QTLs for GS content were mapped to a 3.2-Mb region of chromosome A09, a 50.0-Mb region of chromosome C02, a 39.9-Mb region of chromosome C07, and a 2.8-Mb region of chromosome C09 [18]. We identified two homologous regions that closely resemble the reported intervals on chromosomes A09 and C09, suggesting that they may belong to the same loci. In addition, studies using different rapeseed crosses have mapped the QTLs for GS content on chromosome C02 [4,6,18]. Similarly, the Bn-scaff_17177_1-p546184 marker showing high LD for GS content in 2013 was also found to be located on chromosome C02 in this study. Additionally, the association region close to peak SNPs on A08 is also consistent with the results reported by Xu *et al.* [7]. These results indicate that association mapping is a reliable technique for identifying candidate genes that control the GS content in *B. napus*.

Previous reports have shown that the total GS content is controlled by several genes involved in the GS biosynthesis and transport, and numerous QTLs associated with GS content have been mapped using different populations of *B. napus* [3,4,6,9]. However, candidate genes associated with these major QTLs for GS content in *B. napus* remain to be identified. Recently, the biosynthesis pathways for different glucosinolate compounds were well characterized in *A. thaliana*. These pathways include *GTR1* (*AT3G47960*) and *GTR2* (*AT5G62680*), which are essential for accumulation and allocation of glucosinolates in mature leaves [27] or in the bidirectional distribution of glucosinolates between the roots and rosettes [28,29]. In addition, *MYB34* (*AT5G60890*), *MYB51*, and *MYB122* are reported to coordinately control the suite of enzymes that synthesize indolic glucosinolates [30,31,32,33], but with low transcript levels among different organs of *B. rapa* [34]. *HAG1* (also known as *MYB28*, *AT5G61420*) has been reported to control aliphatic glucosinolate biosynthesis in *A. thaliana* [35]. *MYB28-1* (an isoform of *MYB28*) was involved in aliphatic glucosinolate biosynthesis in *Brassica rapa* (Chinese cabbage) seeds, and the glucosinolate content could be reduced by silencing orthologs of *HAG1* in *B. juncea* [36]. Moreover, both *MYB28* and *MYB34* have been reported to regulate glucosinolate biosynthesis [37]. Here, three *B. napus* homologous of *GRT2*, *MYB28* and *MYB34* were identified according to the reference genome of *B. napus* [38]. Two annotated gene homologues (*AT5G60890*, *MYB34* and *AT5G62680*, *GRT2*) were located within the 2.7–3.0 Mb physical region of *B. napus* chromosome A09, and *AT5G60890* (*MYB34*) was 0.2 Mb away from the SNP Bn-A09-p3029767 and *AT5G62680* was 20 kb away from the significant SNP Bn-A09-p3116738 on chromosome A09, respectively (Table 3). Meanwhile, the candidate gene, *AT5G61420* (referred to as *MYB28* or *HAG1*) was located 0.3 Mb away from the significant SNP Bn-scaff_19783_1-p327775 (Table 3). Therefore, we concluded that *BnGRT2* and *BnMYB28* could be associated with the GS content of *B. napus* (Figure 6). However, these candidate genes are characterized by multigene family members (Table 4). A clear understanding of the relationships among them is necessary for interpreting the functional mechanism of glucosinolate biosynthesis in *B. napus*. Further research will help us to reveal the function of these members of candidate genes in *B. napus*, and these tightly associated SNPs may be used to develop a breeding strategy to improve GS content of oilseed.

## 4. Materials and Methods

### 4.1. Plant Materials and Phenotypic Data

A panel of 520 rapeseed accessions including a wide range of morphological types of various geographical origins were used for association mapping (see Supplementary Material, Table S1 on the journal’s website). All rapeseed accessions were grown in field trials in Beibei (Chongqing, China) in the growing seasons of 2013 and 2014. Each accession was grown at three randomized blocks with three rows and 10–12 plants in each row. Field management essentially followed normal agronomic procedures. Then seeds collected from self-pollinated plants were used for analysis. In brief, the seeds (approximately 3 g seeds per accession) were cleaned of impurity and incased in the circular vessels. Then the glucosinolate content was scanned and calculated by near infrared reflectance spectroscopy (NIRS, FOSS, Hillerød, Denmark) using standard methods. Finally, the mean value from three replicates per accession was calculated for association analysis.

### 4.2. DNA Extraction and SNP Analysis

Genomic DNA was extracted from 100 mg of leaf tissue from 3 young seedlings for each of the 520 rapeseed accessions using the Cetyltrimethylam-monium bromide (CTAB) method. The SNP analysis was performed in the National Key Laboratory of Crop Genetic Improvement, National Subcenter of Rapeseed Improvement in Wuhan, Huazhong Agricultural University, Wuhan, China, according to the manufacture’s protocol [39]. The SNP data were analyzed according to the previous used protocols [18] by Illumina BeadStudio genotyping software.

### 4.3. Population Structure and Relative Kinship Analysis

Population structure, which refers to the non-random distribution of genotypes among individuals within a population, is a key factor affecting the accuracy of association analysis [40]. In this study, population structure evaluation was performed on the 520 accessions using the Bayesian model-based clustering method implemented in STRUCTURE 2.1 [41], and allelic data from the *Brassica* 60K SNP BeadChip Array with 52,157 Infinium Type II SNP loci. The *K*-value (The putative number of genetic groups) best representing the data set was determined according to the method of Evanno *et al.* [42]. In short, three independent runs were performed with a *K*-value varying from *K* = 1 to *K* = 10, with the length of the burn-in period and the number of MCMC (Markov Chain Monte Carlo algorithm) repetitions after burn-in set to 100,000 and 1,000,000, for each of these. Then based on the rate of change in the log probability of data (Ln P (D)) between successive *K*-values, the most likely *K*-value was determined by the log probability of data (*LnP(D)*) and an *ad hoc* statistic ∆*K* as proposed by Evanno *et al.* [42]. TASSEL 5.2.1 [21] was used to estimate the relative kinship coefficients among all pairs of the 520 accessions based on the SNP marker data. Coefficients less than zero were replaced by zero [21].

### 4.4. Genome-Wide Association Study (GWAS) and Candidate Genes Identification

The mixed-model method was developed to account for multiple levels of relatedness simultaneously as detected by random genetic markers [21], and the Q + K model was used to identify the association signals [18]. Therefore, GWAS between SNP markers and seed glucosinolate content of *B. napus* was performed using the mixed-linear model (MLM, Q + K model) implemented in TASSEL 5.2.1, as described by Yu *et al.* [21]. The minimum frequency was set to 0.05, so that only SNP markers with a minor allele frequency (MAF) of 5% or greater were included in the association analysis. The *p*-value, which was determined by the formula *p* = 1/N (where N refers to the total number of SNP markers in study) [18], was used to determine whether SNP markers were significantly associated with the target traits.

In order to identify the candidate genes, the flanking sequences between the adjacent peak SNPs were mapped onto a *B. napus* “Darmor-Bzh” reference genome [38] by a homology search approach via a BLAST analysis. Finally, the *B. napus* genomic sequences centered by the peak SNPs were aligned to the *Arabidopsis* Information Resource [43], and the candidate genes were predicted by BLASTN analysis.

### 4.5. Real-time Quantitative PCR (qRT-PCR) Verification of Candidate Genes

To confirm the association between candidate gene and seed GS contents, total RNA of leaves was extracted from B7, B52 and B95 with relative high GS content and B63, B91 and B342 with low GS content, respectively (see Supplementary Material, Table S1 on the journal’s website). The first cDNA were synthesized using the AMV reverse transcriptase (Takara, Dalian, China). The primers were designed by software Primer Premier 5.0 (see Supplementary Material, Table S2 on the journal’s website) according to the alignment results of candidate gene sequences. Then real-time PCR was performed according to our previous research methods [22]. Relative expressions of candidate genes were calculated with the 2^−ΔΔCt^ method using *BnACTIN7* (At5g09810, EV116054) and *BnUBC21* (At5g25760, EV086936) as internal controls [44]. All samples were amplified in triplicate and the mean value was used for further analysis. All qRT-PCR assays were repeated three times.

## 5. Conclusions

We used association mapping to identify SNP markers linked to glucosinolate content in a population of 520 rapeseed accessions. In total, 11 peak SNPs were repeatedly detected with two years of field data, including four significant association regions distributed on chromosome A08, A09, C03, and C09 of *B. napus*, respectively. Three *B. napus* orthologs of the *Arabidopsis thaliana* genes involved in seed glucosinolate content were identified by comparison of the genomic sequences of adjacent peak SNPs, and *BnGRT2* and *BnMYB28* are the candidate genes involved in the glucosinolate biosynthesis. These results will help us to well understand the molecular mechanism of the glucosinolate biosynthesis and allocation in *B. napus*, and improve the breeding efficiency by molecular marker-assisted selection in the future.

## Supplementary Files

Supplementary File 1
