# Supplementary material for: Identification of Candidate Genes for Seed Glucosinolate Content Using Association Mapping in Brassica napus L
Source: Genes (Basel). 2015 Nov 18;6(4):1215–29. doi: 10.3390/genes6041215 (PMC4690036; doi:10.3390/genes6041215)
Supplement: Supplementary File 1 [file genes-06-01215-s001.pdf]

Supplementary Materials

Table S1. List of rapeseed accessions used in the present study.

Accession No.	Inbred Lines	Country/Region	Seed Glucosinolate Content (μmol·g^{-1})	
			2013	2014
B1	Ganyou 5	Chongqing (China)	102.88	92.57
B2	Nonglin 42	Chongqing (China)	104.38	114.57
B3	Kelina	Chongqing (China)	85.61	77.36
B4	Huaiyou 6	Chongqing (China)	34.02	76.15
B5	Yan 81-2	Chongqing (China)	76.64	60.56
B6	28887	Chongqing (China)	90.83	81.71
**B7**	**Qianyou 4**	**Chongqing (China)**	**108.40**	**110.33**
B8	Qianyou 331	Chongqing (China)	98.58	95.48
B9	Enyou 73-1-2	Chongqing (China)	133.35	129.02
B10	Niuerduo	Chongqing (China)	96.39	110.05
B11	880101	Chongqing (China)	124.10	122.29
B12	SWU40	Chongqing (China)	38.67	28.38
B13	SWU42	Chongqing (China)	32.03	31.88
B14	SWU43	Chongqing (China)	32.53	27.74
B15	SWU44	Chongqing (China)	29.60	25.81
B16	SWU45	Chongqing (China)	29.39	24.23
B17	SWU46	Chongqing (China)	40.13	31.42
B18	SWU47	Chongqing (China)	46.55	43.96
B19	SWU48	Chongqing (China)	41.49	40.97
B20	SWU52	Chongqing (China)	32.79	32.78
B21	SWU53	Chongqing (China)	26.65	25.55
B22	SWU56	Chongqing (China)	40.05	36.54
B23	SWU59	Chongqing (China)	54.57	54.28
B24	SWU65	Chongqing (China)	29.62	26.04
B25	SWU82	Chongqing (China)	38.50	33.61
B26	SWU83	Chongqing (China)	34.84	33.38
B27	SWU92	Chongqing (China)	37.29	31.35
B28	SWU101	Chongqing (China)	38.04	29.30
B29	SWU106	Chongqing (China)	35.94	27.86
B30	SWU108	Chongqing (China)	33.10	47.81
B31	Chuanyou 20	Sichuan (China)	31.78	26.80
B32	Chuanyou 18	Sichuan (China)	29.20	26.58
B33	CY12NY-7	Sichuan (China)	116.12	100.16
B34	CY12Q95406	Sichuan (China)	30.39	28.16
B35	CY12Q8-7	Sichuan (China)	29.63	24.19
B36	CY12QSZ06	Sichuan (China)	27.01	27.01
B37	CY12QCWH-1	Sichuan (China)	40.43	35.37
B38	CY12Q95108	Sichuan (China)	31.80	29.36
B39	CY12Q21535-N3	Sichuan (China)	32.76	41.05
B40	CY12PXW-4	Sichuan (China)	40.61	34.94
B41	CY12PXW-6	Sichuan (China)	34.02	32.50
B42	CY12PXW-9	Sichuan (China)	37.53	32.64
B43	CY13PXW-17	Sichuan (China)	29.60	26.49

Table S1. *Cont.*

Accession No.	Inbred Lines	Country/Region	Seed Glucosinolate Content (μmol·g^{-1})	
			2013	2014
B44	CY14PXW-18	Sichuan (China)	42.37	33.56
B45	CY15PXW-31	Sichuan (China)	35.08	31.60
B46	CY16PXW-35	Sichuan (China)	42.51	40.13
B47	CY17PXW-58	Sichuan (China)	35.24	30.85
B48	CY18PXW-62	Sichuan (China)	34.69	26.14
B49	CY19PXW-65	Sichuan (China)	36.51	39.17
B50	CY20PXW-66	Sichuan (China)	27.34	34.57
B51	CY21PXW-84	Sichuan (China)	35.01	28.15
**B52**	**CY12GJ-1**	**Sichuan (China)**	**102.41**	**105.60**
B53	wx1025	Hunan (China)	39.21	35.22
B54	wx10213	Hunan (China)	39.89	29.97
B55	wx10296	Hunan (China)	39.49	30.91
B56	wx10315	Hunan (China)	28.00	27.09
B57	10-1043	Hunan (China)	34.84	42.39
B58	10-1047	Hunan (China)	33.10	29.75
B59	10-1061	Hunan (China)	40.74	34.81
B60	10-1070	Hunan (China)	42.18	32.53
B61	10-804	Hunan (China)	37.70	43.57
B62	10-1358	Hunan (China)	40.37	32.34
**B63**	**1472**	**Hunan (China)**	**34.74**	**31.58**
B64	Xiangyou 13	Hunan (China)	36.55	32.64
B65	Xiangyou 15	Hunan (China)	31.68	26.62
B66	Xiangyou 11	Hunan (China)	38.07	33.42
B67	740	Hunan (China)	36.22	29.76
B68	631	Hunan (China)	37.86	35.60
B69	613	Hunan (China)	40.88	44.73
B70	783	Hunan (China)	35.81	35.77
B71	782	Hunan (China)	37.67	36.84
B72	YB3	Hunan (China)	36.19	31.24
B73	1360	Hunan (China)	35.35	29.24
B74	563	Hunan (China)	42.55	36.10
B75	WX10329	Hunan (China)	39.24	30.60
B76	santana	Hunan (China)	36.84	34.75
B77	1281	Hunan (China)	43.90	31.24
B78	509	Hunan (China)	34.79	29.98
B79	1368	Hunan (China)	38.59	35.47
B80	1322	Hunan (China)	44.02	28.98
B81	1252	Hunan (China)	35.12	30.90
B82	1321	Hunan (China)	39.52	28.14
B83	07022	Hubei (China)	36.50	30.24
B84	07094	Hubei (China)	33.46	24.89
B85	07016	Hubei (China)	32.60	25.98
B86	9F087	Hubei (China)	36.26	34.81

Table S1. *Cont.*

Accession No.	Inbred Lines	Country/Region	Seed Glucosinolate Content (μmol·g^{-1})	
			2013	2014
B87	97096	Hubei (China)	36.10	37.07
B88	97097	Hubei (China)	33.63	33.39
B89	07189	Hubei (China)	72.04	116.72
B90	07191	Hubei (China)	85.24	102.91
**B91**	**07037**	**Hubei (China)**	**31.30**	**33.99**
B92	RQ011	Hubei (China)	39.60	27.20
B93	RR009	Hubei (China)	32.66	41.98
B94	RR002	Hubei (China)	35.15	26.40
**B95**	**97177**	**Hubei (China)**	**109.00**	**118.18**
B96	96021	Hubei (China)	47.67	54.46
B97	96063	Hubei (China)	44.99	40.03
B98	01111	Hubei (China)	32.84	22.35
B99	01570	Hubei (China)	35.99	29.63
B100	9Bao22	Hubei (China)	47.78	43.61
B101	01188	Hubei (China)	32.91	24.24
B102	02354	Hubei (China)	41.62	39.01
B103	02359	Hubei (China)	33.28	26.68
B104	02365	Hubei (China)	32.58	29.61
B105	93205	Hubei (China)	36.70	32.57
B106	93210	Hubei (China)	36.13	50.84
B107	Nca	Hubei (China)	33.85	33.29
B108	Zhongshuang 4	Hubei (China)	35.46	24.97
B109	Zhongshuang 9	Hubei (China)	32.63	34.64
B110	Zhongshuang 11	Hubei (China)	35.03	23.37
B111	2011-6200	Hubei (China)	24.22	20.53
B112	2011-6308	Hubei (China)	34.23	38.55
B113	2011-7103	Hubei (China)	43.84	38.48
B114	2012-11526	Hubei (China)	34.59	32.04
B115	2012-3448	Hubei (China)	31.62	30.59
B116	2012-3546	Hubei (China)	32.44	26.75
B117	2012-4531	Hubei (China)	33.08	26.87
B118	2012-5086	Hubei (China)	29.48	31.92
B119	2012-5113	Hubei (China)	30.55	30.95
B120	2012-8327	Hubei (China)	32.06	28.03
B121	2012-8355	Hubei (China)	36.12	38.75
B122	2012-8380	Hubei (China)	36.60	30.55
B123	2012-8998	Hubei (China)	34.73	27.79
B124	2012-9323	Hubei (China)	37.66	30.30
B125	2012-9354	Hubei (China)	32.01	31.55
B126	2012-9380	Hubei (China)	27.11	24.92
B127	2012-9478	Hubei (China)	42.53	40.59
B128	2012-9542	Hubei (China)	31.72	30.89
B129	2012-K8053	Hubei (China)	36.66	28.43

Table S1. *Cont.*

Accession No.	Inbred Lines	Country/Region	Seed Glucosinolate Content (μmol·g^{-1})	
			**2013**	**2014**
B130	R2	Hubei (China)	35.20	32.99
B131	Xiwang 106	Hubei (China)	44.05	38.36
B132	Yangguang 198	Hubei (China)	43.12	35.25
B133	Yangguang 2009	Hubei (China)	39.77	32.49
B134	Zhongshuang 10	Hubei (China)	40.76	38.59
B135	Zhongshuang 12	Hubei (China)	33.47	29.32
B136	Zhongshuang 4	Hubei (China)	35.46	23.89
B137	Zhongshuang 6	Hubei (China)	28.99	27.94
B138	Zhongshuang 7	Hubei (China)	34.29	31.04
B139	Zhongyou 589	Hubei (China)	29.23	28.81
B140	Zhongyou 821Q	Hubei (China)	70.59	73.51
B141	Huayou 2	Hubei (China)	78.87	82.71
B142	Major	Hubei (China)	121.82	125.29
B143	Huashuang 2	Hubei (China)	43.53	46.31
B144	Aurora	Hubei (China)	98.65	79.93
B145	Huayou 13	Hubei (China)	120.38	125.58
B146	Rucabo	Hubei (China)	83.79	76.52
B147	Huayou 3	Hubei (China)	86.62	96.06
B148	Huayou 14	Hubei (China)	125.46	128.35
B149	Ningyou 1	Hubei (China)	100.80	112.24
B150	Ceres	Hubei (China)	80.77	70.81
B151	11-9-700	Hubei (China)	88.14	31.06
B152	11-9-701	Hubei (China)	47.11	46.75
B153	11-9-702	Hubei (China)	36.03	28.45
B154	11-9-703	Hubei (China)	41.06	37.31
B155	11-9-704	Hubei (China)	37.95	28.97
B156	11-9-705	Hubei (China)	35.21	30.36
B157	11-9-706	Hubei (China)	42.69	33.49
B158	11-9-707	Hubei (China)	31.37	35.37
B159	11-P63-5Yu7	Hubei (China)	34.88	28.31
B160	11-P63-8Yu32	Hubei (China)	31.72	23.54
B161	11-P63-3Yu3	Hubei (China)	28.94	29.13
B162	11-P67Dong	Hubei (China)	35.34	41.88
B163	09-P64-1	Hubei (China)	36.52	36.02
B164	10-Cong 23	Hubei (China)	33.06	31.03
B165	10-Cong 24	Hubei (China)	39.24	34.72
B166	10-Cong 25	Hubei (China)	31.51	34.99
B167	10-Cong 29	Hubei (China)	38.36	33.29
B168	10-Cong 32	Hubei (China)	39.32	29.80
B169	10-Cong 33	Hubei (China)	40.81	36.32
B170	10-Cong 34	Hubei (China)	32.57	25.63
B171	10-Jiangpeng 2	Hubei (China)	45.40	59.01
B172	10-Jiangpeng 3	Hubei (China)	34.52	29.23

Table S1. *Cont.*

Accession No.	Inbred Lines	Country/Region	Seed Glucosinolate Content (μmol·g^{-1})	
			**2013**	**2014**
B173	11-Yu7-103	Hubei (China)	32.72	30.37
B174	11-Yu7-117	Hubei (China)	38.11	36.44
B175	11-Yu7-125	Hubei (China)	39.27	36.00
B176	11-P74-8	Hubei (China)	36.60	29.71
B177	11-P74-13	Hubei (China)	27.63	23.61
B178	7-7766-74	Hubei (China)	34.31	27.78
B179	P18	Hubei (China)	37.59	31.06
B180	64Peng-10	Hubei (China)	63.27	30.78
B181	Shengguang 77	Hubei (China)	45.89	47.51
B182	Jiayu17peng	Hubei (China)	33.02	33.38
B183	Jiayu25peng	Hubei (China)	41.23	33.63
B184	Jiayu16peng	Hubei (China)	38.49	29.06
B185	Jiayu31peng	Hubei (China)	39.26	28.61
B186	Huashuang5	Hubei (China)	37.71	33.66
B187	Huashuang4	Hubei (China)	34.96	35.10
B188	Jia972	Hubei (China)	39.32	44.23
B189	Huashuang128	Hubei (China)	33.75	29.87
B190	Jia904	Hubei (China)	37.19	30.97
B191	Jia908	Hubei (China)	37.38	38.40
B192	JiaPF190peng	Hubei (China)	38.63	32.43
B193	Jia915	Hubei (China)	33.42	32.10
B194	Jia922	Hubei (China)	31.58	29.05
B195	Jia951peng	Hubei (China)	34.75	30.89
B196	Jia917	Hubei (China)	33.54	29.54
B197	Jia923	Hubei (China)	36.72	28.54
B198	Jia931	Hubei (China)	36.19	53.53
B199	Jiayu05peng	Hubei (China)	29.97	42.52
B200	Jia963peng	Hubei (China)	38.54	27.61
B201	Huyou 17	Shanghai (China)	35.56	27.51
B202	Huyou 15	Shanghai (China)	32.15	26.10
B203	Huyou 12	Shanghai (China)	41.03	27.12
B204	Ningyou 18	Jiangsu (China)	31.42	27.50
B205	Ningyou 16	Jiangsu (China)	37.63	40.52
B206	Ningyou 14	Jiangsu (China)	37.07	33.04
B207	Ningyou 12	Jiangsu (China)	40.05	32.43
B208	Shilijia	Nanjing (China)	66.07	35.98
B209	Shilifeng	Nanjing (China)	55.94	60.19
B210	Yangyou 6	Lixia (Jiangsu, China)	36.81	30.58
B211	Yangyou 5	Lixia (Jiangsu, China)	40.25	35.27
B212	Zhenyou 3	Zhenjiang (China)	44.55	39.66
B213	Hongyou 3	Nanjiang (China)	37.37	30.01
B214	Suyou 1	Jiangsu (China)	34.60	34.82
B215	Zheyou 18	Zhenjiang (China)	33.14	29.74

Table S1. *Cont.*

Accession No.	Inbred Lines	Country/Region	Seed Glucosinolate Content (μmol·g^{-1})	
			**2013**	**2014**
B216	Zheshuang 72	Zhenjiang (China)	44.13	45.57
B217	Zheshuang 8	Zhenjiang (China)	36.99	29.73
B218	Zheyou 758	Zhenjiang (China)	79.90	75.34
B219	Huyou 14	Shanghai (China)	37.33	34.09
B220	Huyou 18	Shanghai (China)	43.67	39.24
B221	Huyou 19	Shanghai (China)	42.67	25.83
B222	Zheyou 19	Zhenjiang (China)	56.73	35.12
B223	Zheyou 21	Zhenjiang (China)	36.59	27.61
B224	Zheshuang 6	Zhenjiang (China)	54.44	47.04
B225	Wanyou 15	Anhui (China)	91.24	96.65
B226	Wanyou 16	Anhui (China)	40.12	42.02
B227	Wanyou 20	Anhui (China)	34.15	33.50
B228	Wanyou 29	Anhui (China)	34.14	27.40
B236	AGREV012	German	30.72	28.36
B237	AGREV019	German	28.97	30.08
B238	AGREV021	German	37.06	37.80
B239	Topas	Sweden	51.28	77.30
B240	cyclon	Denmark	28.08	26.87
B241	Weijie	Canada	34.57	30.45
B242	Sida	Canada	47.49	45.74
B243	Zhizun	Canada	115.22	98.37
B244	Haisheng	Canada	42.14	35.16
B245	D2	Denmark	102.97	85.86
B246	D3	Denmark	51.42	27.70
B247	Qu	United States	36.13	23.57
B248	11-985	Qinghai (China)	49.82	43.65
B249	11-504	Qinghai (China)	34.58	29.81
B250	11-997	Qinghai (China)	43.75	37.55
B251	11-540	Qinghai (China)	32.64	33.51
B252	11-1184	Qinghai (China)	41.11	35.75
B253	10-758	Qinghai (China)	30.40	23.70
B254	10-1230	Qinghai (China)	34.28	34.77
B255	10-847	Qinghai (China)	39.77	31.98
B256	11-1124	Qinghai (China)	42.54	40.91
B257	Shan2A	Shanxi (China)	108.48	113.52
B258	Shan2B	Shanxi (China)	109.70	121.02
B259	KenC1	Shanxi (China)	122.07	109.90
B260	P113	Shanxi (China)	35.06	28.11
B261	P158	Shanxi (China)	29.57	29.21
B262	P310	Shanxi (China)	48.88	39.51
B263	P312	Shanxi (China)	37.66	52.37
B264	P668	Shanxi (China)	36.68	32.97
B265	P685	Shanxi (China)	41.28	36.17

Table S1. *Cont.*

Accession No.	Inbred Lines	Country/Region	Seed Glucosinolate Content (μmol·g^{-1})	
			**2013**	**2014**
B266	A117	Shanxi (China)	45.08	40.66
B268	B250	Shanxi (China)	39.62	37.98
B269	B265	Shanxi (China)	40.68	30.08
B270	A109	Shanxi (China)	131.19	149.83
B271	B285	Shanxi (China)	43.23	40.73
B272	C052	Shanxi (China)	49.07	54.71
B273	GY270	Shanxi (China)	45.66	35.82
B274	GY282	Shanxi (China)	39.05	37.53
B275	GY284	Shanxi (China)	39.53	35.24
B276	B262	Henan (China)	36.61	37.65
B277	A82	Jiangxi (China)	68.34	57.89
B278	A98	Shanxi (China)	85.40	86.82
B279	B414	Xinjiang (China)	111.01	82.02
B280	B308	Sweden	108.65	70.01
B281	A97	Sichuan (China)	95.62	86.35
B282	A148	Sweden	104.58	107.25
B283	B431	Denmark	35.32	37.34
B284	08-P35	Hubei (China)	58.22	41.48
B285	08-P36	Hubei (China)	33.26	29.52
B286	09-P32	Hubei (China)	47.97	51.98
B287	09-P36	Hubei (China)	37.11	28.12
B288	09-P37	Hubei (China)	30.62	28.22
B289	10-P10	Hubei (China)	37.46	39.11
B290	10-P29	Hubei (China)	45.69	46.29
B291	11-P30	Hubei (China)	34.63	29.59
B292	12-P24	Hubei (China)	29.64	37.05
B293	12-P25	Hubei (China)	31.24	29.54
B294	03ⅡB	Gansu (China)	64.96	120.37
B296	03Ⅰ32B	Gansu (China)	48.23	60.13
B297	964	Gansu (China)	78.96	49.54
B298	DDI	Gansu (China)	96.72	68.97
B299	gl302-1	Gansu (China)	117.49	112.42
B300	0679F	Gansu (China)	80.90	62.97
B301	Longyou 2	Gansu (China)	53.47	57.02
B302	03Ⅱ4B	Gansu (China)	108.97	116.96
B303	03LF1	Gansu (China)	50.41	123.86
B304	9852	Gansu (China)	61.76	70.46
B305	9801C	Gansu (China)	67.80	50.64
B306	986	Gansu (China)	64.46	66.84
B307	876	Gansu (China)	76.14	88.26
B308	Wu164	Gansu (China)	59.82	64.28
B309	Longyou 4	Gansu (China)	60.64	82.91
B310	9889	Gansu (China)	57.41	59.34

Table S1. *Cont.*

Accession No.	Inbred Lines	Country/Region	Seed Glucosinolate Content (μmol·g^{-1})	
			**2013**	**2014**
B311	06H7	Gansu (China)	97.69	94.22
B312	Tianyou 4	Gansu (China)	54.53	68.51
B313	Hubeibaihua rapeseed	Hubei (China)	96.81	85.57
B314	Nanchuanchangjiao	Chongqing (China)	51.66	52.50
B315	IMC103	Chongqing (China)	91.18	110.52
B316	Oscar	Chongqing (China)	32.29	29.40
B317	Sophia	German	36.39	27.04
B318	campina	German	31.85	35.49
B319	Conny	German	36.60	35.87
B320	Wesreo	German	109.91	111.99
B321	Wase Chousen	German	132.80	141.35
B322	Gogatsuna	German	134.29	110.86
B323	Nakaee Chousen	German	114.35	108.80
B324	Cat.No.117	German	100.02	115.25
B325	90750	Chongqing (China)	109.42	104.27
B326	Nonglin43	Chongqing (China)	111.59	115.22
B327	Tibet rapeseed	Tibet (China)	99.34	101.67
B328	Youyan2	Chongqing (China)	71.40	87.62
B329	Aijiazao	Wenjiang (Sichuan, China)	111.29	127.74
B330	SWU41	Chongqing (China)	48.21	32.74
B331	SWU49	Chongqing (China)	42.88	40.87
B332	SWU54	Chongqing (China)	111.77	143.58
B333	SWU57	Chongqing (China)	34.25	33.38
B334	SWU60	Chongqing (China)	38.68	40.35
B335	SWU61	Chongqing (China)	31.91	37.59
B336	SWU62	Chongqing (China)	37.51	32.23
B337	SWU63	Chongqing (China)	46.16	33.02
B338	SWU64	Chongqing (China)	40.69	35.33
B339	SWU66	Chongqing (China)	60.20	44.24
B340	SWU67	Chongqing (China)	43.22	39.06
B341	SWU68	Chongqing (China)	32.50	30.09
**B342**	**SWU69**	**Chongqing (China)**	**25.76**	**21.90**
B343	SWU70	Chongqing (China)	36.09	32.89
B344	SWU71	Chongqing (China)	30.85	30.93
B345	SWU74	Chongqing (China)	50.89	38.11
B346	SWU75	Chongqing (China)	37.81	28.97
B347	SWU76	Chongqing (China)	42.55	28.11
B348	SWU77	Chongqing (China)	34.30	36.51
B349	SWU80	Chongqing (China)	39.17	28.27
B350	SWU81	Chongqing (China)	57.19	29.48
B351	SWU84	Chongqing (China)	37.52	28.13
B352	SWU85	Chongqing (China)	41.75	33.89
B353	SWU87	Chongqing (China)	33.72	32.85

Table S1. *Cont.*

Accession No.	Inbred Lines	Country/Region	Seed Glucosinolate Content (μmol·g^{-1})	
			**2013**	**2014**
B354	SWU88	Chongqing (China)	36.98	33.84
B355	SWU89	Chongqing (China)	35.62	25.91
B356	SWU90	Chongqing (China)	36.21	26.99
B357	SWU93	Chongqing (China)	44.57	32.48
B358	SWU94	Chongqing (China)	41.38	38.92
B359	SWU95	Chongqing (China)	109.16	107.01
B360	SWU96	Chongqing (China)	34.11	31.31
B361	SWU99	Chongqing (China)	33.58	31.74
B362	SWU100	Chongqing (China)	44.93	33.87
B363	SWU102	Chongqing (China)	35.70	34.98
B364	SWU103	Chongqing (China)	37.18	35.84
B365	SWU104	Chongqing (China)	41.43	37.96
B366	SWU105	Chongqing (China)	34.09	29.32
B367	SWU107	Chongqing (China)	92.26	58.67
B368	SWU110	Chongqing (China)	38.89	35.65
B369	SWU111	Chongqing (China)	120.87	138.54
B370	SWU112	Chongqing (China)	36.07	30.45
B371	SWU113	Chongqing (China)	45.39	40.41
B372	SWU114	Chongqing (China)	49.01	46.31
B373	Zhongshuang 10	Hubei (China)	80.74	36.21
B374	Zhongshuang 6	Hubei (China)	45.68	34.11
B375	Zhongshuang 7	Hubei (China)	54.91	44.25
B376	Zhongshuang 12	Hubei (China)	50.28	41.38
B377	Zhongyou 589	Hubei (China)	36.96	30.59
B378	Fuyou 4	Hubei (China)	91.35	77.90
B379	Zhen 3736	Hubei (China)	72.00	61.35
B380	Zhen 2609	Jiangsu (China)	70.85	84.30
B381	HX0352	Jiangsu (China)	84.27	92.08
B383	Huyou 21	Shanghai (China)	85.37	98.55
B384	Zheshuang 3	Zhejiang (China)	81.25	100.65
B385	Zheyou 21	Zhejiang (China)	66.11	63.51
B386	Wanyou 20	Anhui (China)	73.66	90.61
B387	Wanyou 12	Anhui (China)	68.63	89.10
B388	Wanyou 7	Anhui (China)	87.21	91.48
B389	Hongyou 3	Nanjing (China)	79.48	55.06
B390	Zhenyou 5	Hubei (China)	91.83	92.70
B391	Yangyou 4	Hubei (China)	87.57	88.32
B392	Huyou 15	Shanghai (China)	76.21	84.72
B393	Huyou 16	Shanghai (China)	96.94	83.19
B394	Huyou 17	Shanghai (China)	64.17	43.54
B395	Huyou 18	Zhejiang (China)	88.03	65.15
B396	Zheshuang 72	Zhejiang (China)	79.15	50.05
B397	Zheshuang 8	Zhejiang (China)	74.94	64.19

Table S1. *Cont.*

Accession No.	Inbred Lines	Country/Region	Seed Glucosinolate Content (μmol·g^{-1})	
			**2013**	**2014**
B398	Zheyou 50	Zhejiang (China)	81.00	64.99
B399	Suyou 1	Jiangsu (China)	37.73	29.43
B400	Zhongshuang 4	Hubei (China)	35.08	28.17
B401	Zhongshuang 11	Hubei (China)	35.05	30.05
B402	Yangguang 198	Hubei (China)	31.17	26.55
B403	Huahang 901	Hubei (China)	44.24	38.00
B404	Yang J6711	Jiangsu (China)	42.57	33.19
B405	Yan 6055	Jiangsu (China)	43.73	32.03
B406	Yangjian 8	Jiangsu (China)	37.65	35.58
B407	Xiwang 106	Hubei (China)	41.08	34.04
B408	Zheyou 17	Zhejiang (China)	45.19	32.31
B409	Zhongshuang 5	Hubei (China)	45.40	80.78
B410	Zhongyou 821	Hubei (China)	33.69	28.18
B411	Qinyou 1	Hubei (China)	145.24	136.64
B412	Weilong 88	Hubei (China)	46.41	30.01
B413	Yanyou 2	Hubei (China)	38.82	38.67
B414	Qinyou 5	Hubei (China)	37.16	37.26
B415	Suyou 4	Jiangsu (China)	38.00	37.71
B416	Fengyou 9	Hubei (China)	40.05	30.27
B417	De 68-12	Hubei (China)	38.41	34.75
B418	Monty	Hubei (China)	38.77	57.66
B419	Oscar	Hubei (China)	35.64	48.99
B420	Ningyou 12	Hubei (China)	52.68	61.08
B421	Ningyou 14	Hubei (China)	47.47	32.15
B422	Shilifeng	Hubei (China)	72.56	31.50
B423	Ningyou 18	Hubei (China)	49.44	31.48
B424	Ningyou 10	Hubei (China)	50.22	21.52
B425	Helios	Hubei (China)	59.44	59.64
B426	Hector	Hubei (China)	123.45	115.18
B427	Mian 96—203 (09)	Qinghai (China)	41.72	77.08
B428	Qing 662A	Hubei (China)	104.45	71.31
B429	699	Hubei (China)	36.82	40.16
B430	Canada 2	Hubei (China)	106.27	127.94
B431	Zhongshuang 2	Hubei (China)	32.26	27.46
B432	Zhongshuang 9	Hubei (China)	37.35	27.77
B433	WH-12	Hubei (China)	40.58	30.28
B434	WH-15	Hubei (China)	43.77	31.35
B435	WH-17	Hubei (China)	36.64	30.99
B436	WH-19	Hubei (China)	35.94	27.95
B437	WH-20	Hubei (China)	37.91	29.89
B438	WH-23	Hubei (China)	30.75	34.06
B439	WH-24	Hubei (China)	35.36	29.13
B440	WH-25	Hubei (China)	36.11	30.02

Table S1. *Cont.*

Accession No.	Inbred Lines	Country/Region	Seed Glucosinolate Content (μmol·g^{-1})	
			**2013**	**2014**
B441	WH-26	Hubei (China)	34.94	48.68
B442	WH-27	Hubei (China)	33.59	28.47
B443	WH-28	Hubei (China)	30.47	28.22
B444	WH-29	Hubei (China)	38.60	41.91
B445	WH-30	Hubei (China)	37.27	32.35
B446	WH-31	Hubei (China)	35.05	28.42
B447	WH-33	Hubei (China)	35.20	30.36
B448	WH-37	Hubei (China)	56.05	40.94
B449	WH-38	Hubei (China)	46.36	33.04
B450	WH-41	Hubei (China)	43.71	30.93
B451	WH-42	Hubei (China)	52.34	42.51
B452	WH-43	Hubei (China)	42.76	37.22
B453	WH-45	Hubei (China)	42.15	36.74
B454	WH-49	Hubei (China)	37.29	28.98
B455	WH-50	Hubei (China)	39.35	67.87
B456	WH-55	Hubei (China)	45.96	38.53
B457	WH-56	Hubei (China)	34.75	40.50
B458	WH-57	Hubei (China)	36.84	32.67
B459	WH-58	Hubei (China)	32.05	29.27
B460	WH-59	Hubei (China)	41.57	39.27
B461	WH-60	Hubei (China)	35.53	47.37
B462	WH-61	Hubei (China)	35.47	25.38
B463	WH-62	Hubei (China)	38.73	30.12
B464	WH-63	Hubei (China)	35.65	28.63
B465	WH-81	Hubei (China)	45.49	26.66
B466	WH-83	Hubei (China)	31.97	26.66
B467	WH-85	Hubei (China)	48.25	35.49
B468	WH-88	Hubei (China)	34.28	22.74
B469	WH-93	Hubei (China)	39.28	38.54
B470	WH-95	Hubei (China)	33.26	31.95
B471	WH-100	Hubei (China)	31.98	28.12
B472	WH-127	Hubei (China)	40.43	35.53
B473	Yuyou1	Henan (China)	29.25	24.37
B474	COBRA	Hubei (China)	81.56	48.12
B475	NY7	Hubei (China)	91.33	91.79
B476	Tapidor	Hubei (China)	65.33	123.07
B477	Huayou 6	Hubei (China)	115.53	93.18
B478	Huayou 12	Hubei (China)	37.80	29.69
B479	Cubs root	Hubei (China)	102.31	90.11
B480	Huayou 10	Hubei (China)	101.37	96.50
B481	Bienvenu	Hubei (China)	117.54	126.41
B482	Shengli rapeseed	Hubei (China)	99.43	97.99
B483	ERAKE	Hubei (China)	39.23	35.51

Table S1. *Cont.*

Accession No.	Inbred Lines	Country/Region	Seed Glucosinolate Content (μmol·g^{-1})	
			2013	2014
B484	Taisetsu	Hubei (China)	109.34	109.99
B485	cresor	Hubei (China)	93.61	96.09
B486	Daichousen	Hubei (China)	85.18	73.96
B487	comet	Hubei (China)	40.58	51.78
B488	Niklas	Hubei (China)	124.59	121.72
B489	Askari	Hubei (China)	121.35	140.95
B490	chuosenshu	Hubei (China)	93.42	110.22
B491	WESBROOK	Hubei (China)	50.00	41.60
B492	Suigenshu	Hubei (China)	114.96	104.62
B493	Huayou 4	Hubei (China)	125.30	132.35
B494	Shengliqinggeng	Shanghai (China)	91.80	74.23
B495	Mijiaoduotou rapeseed	Shanghai (China)	105.53	115.36
B496	Aijishengli	Shanghai (China)	101.17	97.69
B497	Caojingshengli	Shanghai (China)	115.39	126.19
B498	Huyou 3	Shanghai (China)	106.66	121.73
B499	Shaoyeqing	Shanghai (China)	91.75	89.83
B500	African rapeseed ruhuanghua	Shanghai (China)	135.65	162.51
B501	Hujizao	Shanghai (China)	77.31	77.18
B502	Caoyou 2	Shanghai (China)	119.34	132.07
B503	Huyou 16	Shanghai (China)	48.85	41.08
B504	Fengding 240	Jiangsu (China)	95.29	102.30
B505	Quanzi rapeseed	Jiangsu (China)	35.02	45.02
B506	Dahuaqiu	Jiangsu (China)	105.21	109.36
B507	Rongxuan	Jiangsu (China)	72.09	70.76
B508	Ningyou 10	Jiangsu (China)	120.41	141.82
B509	Ningyou 8	Jiangsu (China)	103.32	75.93
B510	Ningyou 6	Jiangsu (China)	99.12	102.92
B511	Duoyou 1	Jiangsu (China)	103.97	96.19
B512	Huaiyou 6	Jiangsu (China)	105.52	113.64
B513	Huaiyou 12	Jiangsu (China)	113.28	119.54
B514	Peixuan 170	Jiangsu (China)	99.81	84.64
B515	Guangde 138	Anhui (China)	110.45	109.62
B516	Guangde 8104	Anhui (China)	125.04	137.73
B517	Dangyouzao 1	Anhui (China)	96.69	67.27
B518	Guangde 761	Anhui (China)	108.31	106.39
B519	Tonglinghuaye	Anhui (China)	128.19	133.44
B520	Chu 610	Anhui (China)	111.54	111.68
B521	Chu 107	Anhui (China)	117.22	141.73
B522	Su 84-6	Anhui (China)	117.66	109.86
B523	Huanyouzao	Anhui (China)	103.22	103.75
B524	Chuyou1	Anhui (China)	106.52	101.26
B525	Chuxianbaihua	Anhui (China)	99.29	117.09
B526	Jie 65-1	Zhejiang (China)	114.59	105.62

Table S1. *Cont.*

Accession No.	Inbred Lines	Country/Region	Seed Glucosinolate Content (μmol·g^{-1})	
			**2013**	**2014**
B527	Shenhuang1	Shanghai (China)	68.99	66.45
B528	Zheyou 601	Zhejiang (China)	36.77	31.44
B529	Sangao rapeseed	Zhejiang (China)	38.95	25.95
B530	Zaofeng 1	Zhejiang (China)	129.91	142.25

Table S2. Primer information used for qRT-PCR analysis between the high and low seed glucosinolate content *B. napus*.

Primers	Forward Sequence (5'-3')	Reverse Sequence (5'-3')	Length (bp)	Tm (°C)
BnGTR2-1	AAGACTCTTACCGTTGCGGTCCTC	TCCCTCCTGCTCCCACGACA	184	64
BnGTR2-2	GGGTTAAAGCCAACAAAAGAGCCT	TCAAGAAGACAACGTAAGTGGCTGC	364	60
BnGTR2-3	ACACCCAACAATTCAGGTTTCTCG	ACGTTCCAAGACACGGTCATAAAAT	335	64
BnMYB34-1	TCCGGTTCAGCACGGGTTCT	CTTCGTTGCTAAAGAACTCGGTGA	256	60
BnMYB34-2	CGTCGCCGACGTTTCACAAAACTCC	ATCAGACAAAGAACAGTTGTCGGAG	155	62
BnMYB28	GGACCACCGAGGAAGATAAGAAACT	CGTTTCTTGAGATGTGTGTTCCAGT	295	62

© 2015 by the authors; licensee MDPI, Basel, Switzerland. This article is an open access article distributed under the terms and conditions of the Creative Commons Attribution license (http://creativecommons.org/licenses/by/4.0/).
